# Application of convolutional neural network in fusion and classification of multi-source remote sensing data

**DOI:** 10.3389/fnbot.2022.1095717

**Published:** 2022-12-22

**Authors:** Fanghong Ye, Zheng Zhou, Yue Wu, Bayarmaa Enkhtur

**Affiliations:** ^1^Land Satellite Remote Sensing Application Center, Ministry of Natural Resources of People's Republic of China, Beijing, China; ^2^School of Resource and Environmental Sciences, Wuhan University, Wuhan, China; ^3^Ecology and Environment Monitoring and Scientific Research Center, Ministry of Ecology and Environment of the People's Republic of China, Wuhan, China; ^4^Department of Natural Resources of Heilongjiang Province, Heilongjiang Provincial Institute of Land and Space Planning, Harbin, China; ^5^Geospatial Information and Technology Department, Agency for Land Administration and Management, Geodesy and Cartography, Ulaanbaatar, Mongolia

**Keywords:** remote sensing image, convolutional neural network, double branch structure, hyperspectral, DB-CNN algorithm, lidar data

## Abstract

**Introduction:**

Through remote sensing images, we can understand and observe the terrain, and its application scope is relatively large, such as agriculture, military, etc.

**Methods:**

In order to achieve more accurate and efficient multi-source remote sensing data fusion and classification, this study proposes DB-CNN algorithm, introduces SVM algorithm and ELM algorithm, and compares and verifies their performance through relevant experiments.

**Results:**

From the results, we can find that for the dual branch CNN network structure, hyperspectral data and laser mines joint classification of data can achieve higher classification accuracy. On different data sets, the global classification accuracy of the joint classification method is 98.46%. DB-CNN model has the highest training accuracy and fastest speed in training and testing. In addition, the DB-CNN model has the lowest test error, about 0.026, 0.037 lower than the ELM model and 0.056 lower than the SVM model. The AUC value corresponding to the ROC curve of its model is about 0.922, higher than that of the other two models.

**Discussion:**

It can be seen that the method used in this paper can significantly improve the effect of multi-source remote sensing data fusion and classification, and has certain practical value.

## 1. Introduction

As a depth detection technology, remote sensing is applied to space exploration, urban planning, rescue and disaster relief. It combines multi-disciplinary technologies such as earth science, space science, and computer, so it has different characteristics in terms of scope of use and technical tools (Demir and Ulke, 2020; Zhou et al., 2021c; Du et al., 2022; Lu et al., 2022). However, facing different application scenarios, remote sensing image classification needs higher accuracy, and the accuracy and performance of image classification determine the quality of the application effect. Remote sensing images usually contain a lot of spectral information, which can be used in image recognition and classification (Hu et al., 2021). In remote sensing, classification and recognition of related images is an important function, and different classification and recognition methods have different effects (Yu, 2020). The previous classification methods can not classify well, and the classification results are poor. The classification technology based on the deep learning algorithm has been studied by many scholars because of its high classification effect and performance. Convolutional neural network (CNN) has shown good performance in image feature extraction and classification. In this paper, it is applied to remote sensing image classification to improve its classification accuracy and performance.

## 2. Related work

In the study of remote sensing images, the main content focuses on the fusion and classification of remote sensing data. During this period, different scholars adopted different research methods. For example, Du et al. (2021) applied methods such as integrated hyperspectral images to extract and analyze remote sensing image features. After verification, it is found that the proposed method can achieve effective classification (Du et al., 2021). In the process of classifying multi-source remote sensing data, Pastorino et al. (2021) designed a hierarchical probabilistic graphical model, which combines Markov framework and decision tree method, which has certain effectiveness and feasibility (Pastorino et al., 2021). In order to improve the classification effect of remote sensing images, Luo et al. (2021) designed a combination strategy based on sorting batch mode, combined with spectral information divergence, and good classification effect can be obtained (Luo et al., 2021). Dong R. et al. (2020) proposed a fast depth-aware network that combines multiple advantages to achieve simultaneous extraction of deep and shallow features (Dong R. et al., 2020). Zhang and Han (2020) used the multi-target classification recognition model when carrying out remote sensing image segmentation and feature extraction. Through correlation verification, it can better perform correlation recognition and has strong robustness (Zhang and Han, 2020). Bazi et al. (2021) proposed a remote sensing image classification model based on the vision converter, in which the context relationship is represented through the multi head attention mechanism. After relevant verification, it is found that the classification effect of this method is better (Bazi et al., 2021). In the process of remote sensing image classification, there will be a problem of data feature distortion. Face this problem, Dong Y. et al. (2020) designed a spectral space weighted popular embedded distribution alignment method, and proved its effectiveness and practical value through experiments (Dong Y. et al., 2020). On the basis of multi-scale feature fusion, Zhang C. et al. (2020) proposed the corresponding remote sensing image classification method, which uses a new weighted eigenvalue convolutional neural network to segment images, and achieved good experimental results (Zhang C. et al., 2020). Xu Y. et al. (2019) analyzed the data fusion contest held in 2018, summarized a variety of multi-source optical remote sensing, analyzed its related land cover classification applications, and the machine vision algorithms involved. The effective combination of machine learning and observation data has become a good data analysis method (Xu Y. et al., 2019). Jin and Mountrakis (2022) classified the land cover types through the random forest algorithm, during which the remote sensing data sources were involved. The results show that the highest overall accuracy of the algorithm is 83.0%, which is much higher than the accuracy of other sensors (Jin and Mountrakis, 2022).

Ma et al. (2020) used improved CNN to classify seismic remote sensing images, and verified the method. After verification, it can have a high accuracy, and its excellent performance has an important role in earthquake prevention and disaster relief (Ma et al., 2020). Pan et al. (2020) corrected the high-resolution remote sensing classification results through end-to-end localization post-processing. This method can achieve effective correction and make the classification results have high accuracy (Pan et al., 2020). Han et al. (2020) designed a classification method combining 3D-CNN and squeeze excitation network to classify relevant sea ice remote sensing images. The practical value of this method has been proved through relevant research (Han et al., 2020). Qing et al. (2021) designed an end-to-end Transformer model and applied it to hyperspectral image classification, and the experimental results showed that it has high performance (Qing et al., 2021). Sun et al. (2021) designed a ConvCRF model with boundary constraints, which was used to improve the classification method of synthetic aperture radar images, thereby improving the classification accuracy of remote sensing images (Sun et al., 2021). Samat et al. (2020) improved the extreme gradient boosting (XGBoost) algorithm and proposed a Meta-XGBoost algorithm, which integrated the advantages of multiple methods and improved the effect of hyperspectral remote sensing image classification (Samat et al., 2020). He et al. (2020) combined a fully convolutional network with a popular graph embedding model and applied it to PolSAR image classification, which proved to have high application performance (He et al., 2020).

The above studies have used different deep learning methods to classify and identify different types of remote sensing images, and have achieved good application results. Although some methods can achieve good experimental results, the experimental process is more complicated, so there is still room for improvement in efficiency. The research adopts CNN based classification method, which can classify efficiently and has high classification accuracy.

## 3. Multi-source remote sensing data fusion and classification based on CNN

### 3.1. Build CNN model

**Figure 1:**
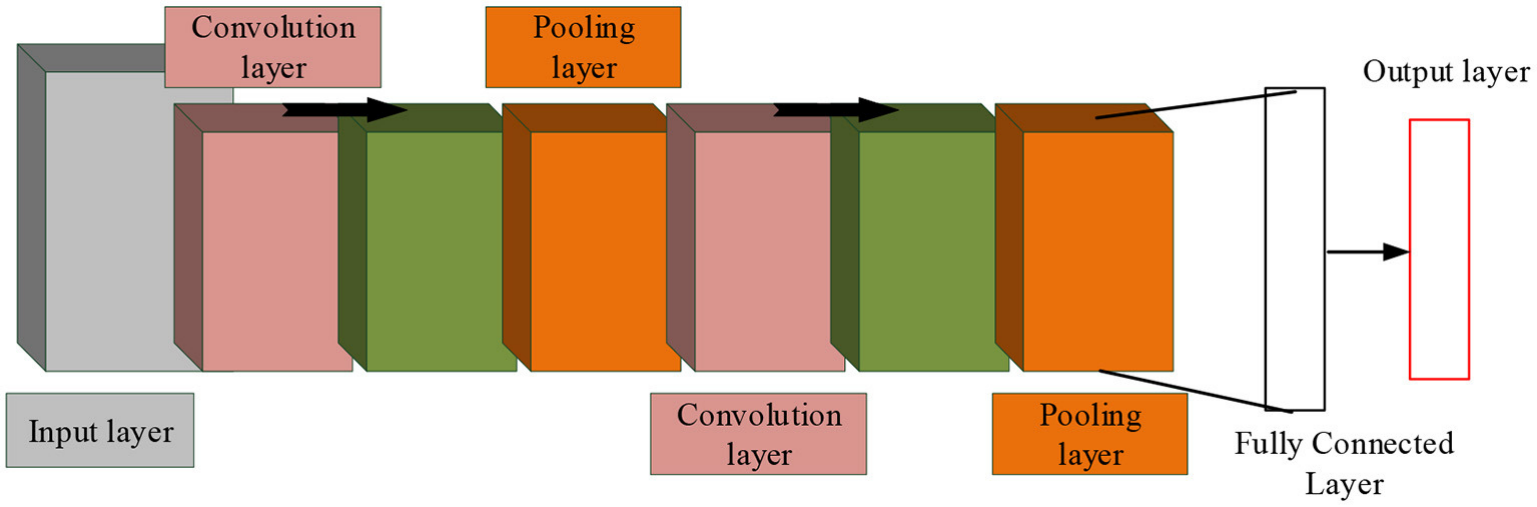
Convolutional neural network structure diagram.

With the continuous progress of remote sensing technology, the application scope of remote sensing image data is expanding. The application of remote sensing image data is conducive to better urban planning. Before that, it is necessary to classify multi-source remote sensing data to perform other operations. CNN algorithm has strong feature extraction ability and is widely used in data classification. Therefore, CNN is applied in multi-source remote sensing data fusion classification. As a feedforward neural network, CNN includes convolution structure and multilayer non-linearity. The algorithm can extract middle and high level abstract features from remote sensing images under the action of convolution layer and pooling layer (Deng et al., 2020; Huang et al., 2022; Zhong et al., 2022; Zhou et al., 2022). The convolutional neural network represents the target by building a multi-layer network, and its structure is shown in Figure 1.

In Figure 1, CNN includes multiple layers, such as convolution layers. At the same time, in this algorithm, features can be extracted and classified. In a convolutional neural network, each image can be represented by a matrix of pixel values. Meanwhile, in the convolution layer, the neurons are connected in a special way, and the image edges and features are extracted (Zhang et al., 2020). And the convolution operation can process image noise, and can also enhance some features. Under complex conditions, through the action of activation function, the non-linear ability of the network is strengthened. For the binary classification problem, the Sigmoid function is used, while for the image recognition classification, the ReLU function is used (Chung et al., 2020; Zhou et al., 2021a,b; Zhang et al., 2022). Finally, the model needs to be downsampled to reduce its complexity, which is done through a pooling operation. The fully connected layer belongs to the classification and recognition part, which performs weighted summation of the extracted features and performs the final output. As a key part of the convolutional neural network, the convolution layer mainly performs feature extraction and dimensionality reduction processing operations. It contains many convolution kernels, which convolve with the input and generate new feature maps. Convolution usually contains both single-channel and multi-channel types (Feng et al., 2021). Among them, the one-dimensional convolution usually plays the role of signal processing. Assuming that the input signal is listed as *x*_*t*_, and *t* = 1, 2, ⋯ , *n*, then its output expression is shown in Formula (1).


(1) $$\begin{array}{l} y_{t}=\overset{K}{\underset{k=1}{\sum }}w_{k}x_{t-k+1} \end{array}$$

In Formula (1), *w*_*k*_ is the convolution kernel, and *K* is the length of the convolution kernel. In the processing of images and videos, two-dimensional convolution is used more frequently. Let the 2D image input be *x*_*ij*_, where 1 ≤ *i* ≤ *M*, 1 ≤ *j* ≤ *N*. In the same way, *w*_*ij*_ represents the convolution kernel, where 1 ≤ *i* ≤ *m*, 1 ≤ *j* ≤ *n*. Then its output expression is shown in Formula (2).


(2) $$\begin{array}{l} y_{ij}=\overset{m}{\underset{u=1}{\sum }}\overset{n}{\underset{v=1}{\sum }}w_{uv}x_{i-u+1,j-v+1} \end{array}$$

**Figure 2:**
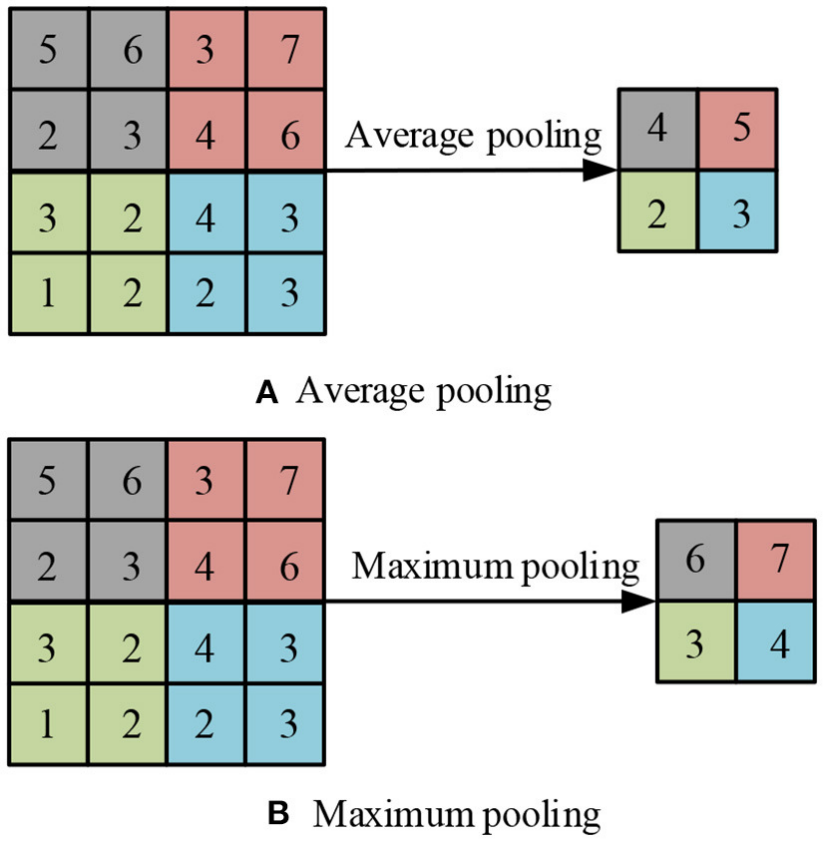
Average pool and maximum pool structure.

In Formula (2), *w*_*uv*_ is the convolution kernel, and *m, n* is the length of the convolution kernel. In Formula (2), we know that during the convolution operation, the filter remains stable and the entire input part is processed. At the same time, the convolution process can be trimmed by changing the step size and padding, which has a certain adjustment effect on the sliding amplitude, thereby making the boundary more complete. The pooling layer is a non-linearly connected area, located between convolution layers, and its adjacent layers are connected to each other through neurons. When extracting the main features of the image, the pooling layer has a good performance. First, the pooling layer can effectively reduce the amount of computation, thereby saving resources. Second, the pooling layer can reduce the number of parameters and the complexity of the model, thereby avoiding overfitting and ensuring scale and space invariance (Li et al., 2020). Average pooling and max pooling are the two most common methods of pooling operations, which can effectively retain the original image features. The structure diagram is shown in Figure 2.

In Figure 2, these two operations can reduce the error of feature extraction, the variance of estimated value caused by the domain, and the shift of estimated mean value caused by the error of convolution parameters. After two operations, activate the data through the activation function, which is a key step in CNN. Neural networks are generally linear calculations, and complex functions are not generated during the calculation process. The activation function can add complex models to it and effectively enhance the non-linear expression ability of the network. These functions of the activation function can play a good role in solving complex network problems, while improving the fitting ability of the model. Common activation functions are Sigmoid, Tanh, and ReLU. Among them, the definition of the sigmoid activation function is shown in Formula (3).


(3) $$\begin{array}{l} Sigmoid\left(z\right)=\frac{1}{1+e^{-z}} \end{array}$$

In Formula (3), the output value of the sigmoid activation function is between (0, 1) and has monotonicity. Its image is similar to the sigmoid, which has the advantage of stable optimization. The definition of the Tanh activation function is shown in Formula (4).


(4) $$\begin{array}{l} \mathrm{Tanh}\left(z\right)=\frac{e^{z}-e^{-z}}{e^{z}+e^{-z}} \end{array}$$

In Formula (4), the output value of the Tanh activation function is between (−1, 1) and is centered at 0. At the same time, its image curve is also similar to the S-shape, and the convergence speed is faster. The relevant expression of ReLU activation function is Formula (5).


(5) ReLU(z)=max(0,z)

In Formula (5), when the input value is positive, the derivative of the function is always 1. Therefore, compared with the Sigmoid activation function and the Tanh activation function, it has a faster calculation speed and can effectively save resources. After the above operations are completed, the data is normalized to eliminate the influence of the index on the value. In the normalization processing operation, Faced with the problems of slow convergence speed and scattered characteristics, it is necessary to process each batch of data. For the same batch of data *X*_*B*_ = {*x*_1_, *x*_2_⋯ , *x*_*n*_}, the mean and variance expressions are shown in Formula (6) and Formula (7).


(6) $$\begin{array}{lll} \mu_{B} & = & \frac{1}{m}\overset{m}{\underset{i=1}{\sum }}x_{i} \end{array}$$

(7) $$\begin{array}{lll} \sigma_{B}^{2} & = & \frac{1}{m}\overset{m}{\underset{i=1}{\sum }}{\left(x_{i}-\mu_{B}\right)}^{2} \end{array}$$

In Formula (6) and Formula (7), μ_*B*_ and $\sigma_{B}^{2}$ are the mean and variance, respectively, and a new mapping ${\hat{x}}_{i}$ can be obtained after normalization *x*_*i*_, and its expression is shown in Formula (8).


(8) $$\begin{array}{l} {\hat{x}}_{i}=\frac{x_{i}-\mu_{B}}{\sqrt{\sigma_{B}^{2}+\varepsilon }} \end{array}$$

In Formula (8), ε > 0 and the value is smaller. In order to obtain the real and effective distribution of network data, scale transformation and offset processing are added after normalization, and its expression is shown in Formula (9).


(9) $$\begin{array}{l} y_{i}=\gamma {\hat{x}}_{i}+\beta \end{array}$$

In Formula (9), γ and β are parameters in network training, and the update methods are shown in Formula (10) and Formula (11).


(10) $$\begin{array}{lll} \nabla \gamma & = & \overset{m}{\underset{i=1}{\sum }}\nabla y_{i}\frac{\partial y_{i}}{\partial y}=\overset{m}{\underset{i=1}{\sum }}\nabla y_{i}\cdot {\hat{x}}_{i} \end{array}$$

(11) $$\begin{array}{lll} \nabla \beta & = & \overset{m}{\underset{i=1}{\sum }}\nabla y_{i}\frac{\partial y_{i}}{\partial \beta }=\overset{m}{\underset{i=1}{\sum }}\nabla y_{i}\cdot 1=\overset{m}{\underset{i=1}{\sum }}\nabla y_{i} \end{array}$$

In Formula (10) and Formula (11), the two are updated by means of derivation, and the input *x*_*i*_ gradient expression is shown in Formula (12).


(12) $$\begin{array}{l} \nabla x_{i}=\nabla \hat{x}\cdot \frac{1}{\sqrt{\sigma_{B}^{2}+\varepsilon }}+\nabla \sigma_{B}^{2}\cdot \frac{2\left(x_{i}-\mu_{B}\right)}{m}+\nabla \mu_{B}\cdot \frac{1}{m} \end{array}$$

In Formula (12), there is a certain relationship between *x*_*i*_, ${\hat{X}}_{I}$, μ_*B*_ and $\sigma_{B}^{2}$. At the same time, in the back-propagation process, calculate the gradient of ${\hat{X}}_{I}$, μ_*B*_ and $\sigma_{B}^{2}$ to *x*_*i*_, as shown in Formula (13), Formula (14), and Formula (15).


(13) $$\begin{array}{lll} \nabla {\hat{x}}_{i} & = & \nabla y_{i}\cdot \gamma \end{array}$$

(14) $$\begin{array}{l} \nabla \mu_{B}=\sum_{i=1}^{m}\nabla \hat{x}\cdot \frac{-1}{\sqrt{\sigma_{B}^{2}+\varepsilon }}+\nabla \sigma_{B}^{2}\cdot \\ \;\frac{1}{m}\sum_{i=1}^{m}-2\left(x_{2}-\mu_{B}\right) \end{array}$$

(15) $$\begin{array}{lll} \nabla \sigma_{B}^{2} & = & \overset{m}{\underset{i=1}{\sum }}\nabla \hat{x}\cdot \left(x_{i}-\mu_{B}\right)\cdot \frac{-1}{2}{\left(\sigma_{B}^{2}+\varepsilon \right)}^{-\frac{3}{2}} \end{array}$$

After the feature extraction and classification and recognition are completed, the results are output, thus completing the entire convolutional neural network steps.

### 3.2. Multi source remote sensing data fusion and classification based on CNN

**Figure 3:**
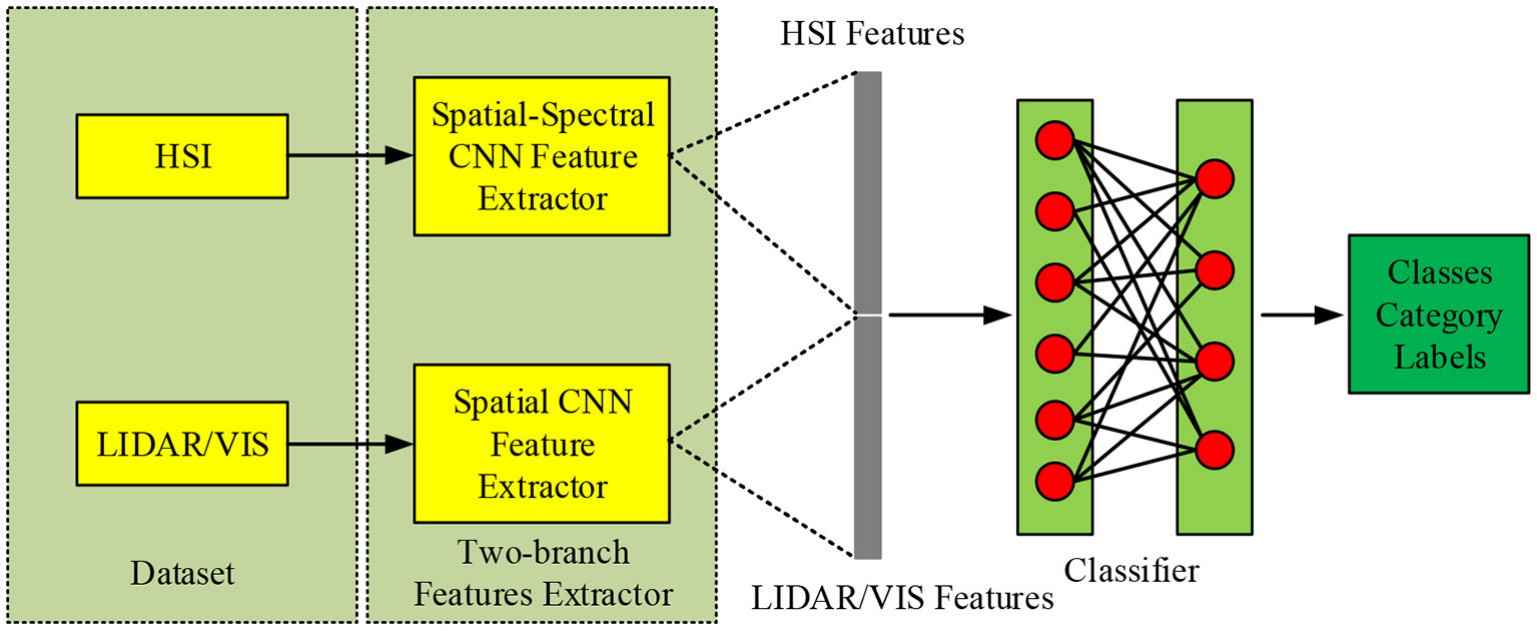
Related flow chart.

Multi-source remote sensing data includes hyperspectral data (HSI) and lidar data (LiDAR), due to their different types and applicable directions, there are certain challenges in fusion and classification (Qu et al., 2021). Therefore, the research uses CNN to extract its features, and proposes a dual-branch convolutional neural network (DB-CNN), which is convenient for organically combining multiple data sources. The multi-source remote sensing data fusion and classification process based on CNN is shown in Figure 3.

In Figure 3, a dual-channel CNN network is used to extract spectral information. In HSI branch, Conv2D3 of 2-D channel is 256, Conv2D3 is 512, Max Pool is 2 ^*^ 2, Conv1D11 of 1-D channel is 256, Conv1D3 is 512, Max Pool is 2 ^*^ 1; In the HSI branch, the value of Conv2D3 is 64, the value of Cascade2D is [128, 64,128, 64], the value of Max Pool is 2 ^*^ 2, and the value of Cascade2D is [256128256128]. For hyperspectral data extraction, the spatial information is extracted by 2-D CNN, and the central pixel information is extracted by 1-D CNN. For LiDAR and Visible Light Image (VIS) data, because of their strong spatial information, the same network can be used for feature extraction. The overall network structure consists of three parts, namely spectrum, spatial channel and space-spectral fusion. The spectral channel can be divided into three parts, including convolution layer, pooling layer, etc., and batch normalization. When performing the convolution operation, a one-dimensional convolution method is adopted to process the one-dimensional vector of the spectral data. At the same time, in order to correct the data distribution, the Leaky ReLU activation function is selected to perform the correction operation. Therefore, the spectral dimension feature extraction process can be expressed as: firstly, input the spectral vector $H_{ij}^{spec}$ into the network, then, perform correlation operation through it, and finally output the feature $F_{ij}^{spec}$, and expand the feature into a one-dimensional vector at the same time.

For spatial dimension feature extraction, the processing object is usually *r* the image block with radius around the center pixel, so the output feature $F_{ij}^{spat}$ is the information of the center pixel and its surrounding radius *r*. It will also expand $F_{ij}^{spat}$ into a one-dimensional vector and fused with $F_{ij}^{spec}$ each other. When extracting relevant features, the consistency of the depth and structure of the dual channel network shall be ensured to make the extracted features more complete. The two kinds of features are fed into the fully connected layer after fusion, and they are reorganized and selected by learning. For the features with too little contribution, the Dropout method can be used to discard them, and the whole process can be represented by Formula (16).


(16) $$\begin{array}{l} T\left(F_{ij}^{spat},F_{ij}^{spec}\right)=f\left(W\left(F_{ij}^{spat}||F_{ij}^{spec}\right)+b\right) \end{array}$$

Formula (16), ▪||▪ denote feature fusion, *W* and *b* denote the weights and biases of fully connected layers. Then the above formula can be expressed as *F*_*hsi*_ and input into the softmax classifier. The classifier can predict features as corresponding probability distributions, as shown in Formula (17).


(17) $$\begin{array}{l} pred\left(i,j\right)=\\ \frac{1}{\sum_{n=1}^{C}\left(\exp\left({\theta^{\prime }}_{n}F_{hsi}\right)\right)}\left[\begin{array}{c} \exp\left({\theta^{\prime }}_{1}F_{hsi}\right)\\ \exp\left({\theta^{\prime }}_{2}F_{hsi}\right)\\ \vdots \\ \exp\left({\theta^{\prime }}_{C}F_{hsi}\right) \end{array}\right] \end{array}$$

**Figure 4:**
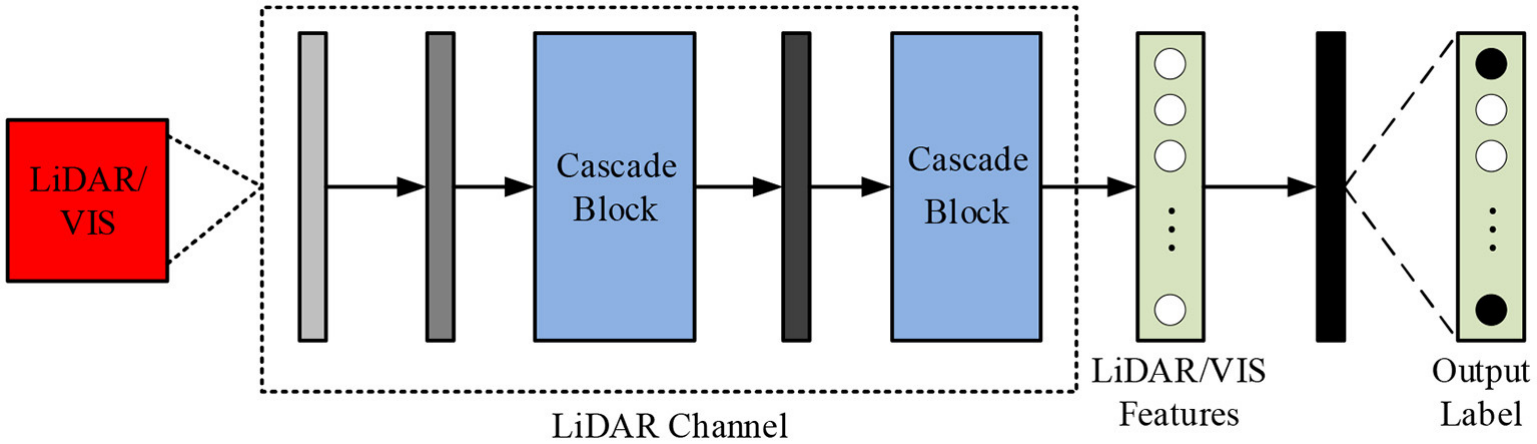
CNN network structure of cascaded modules for LiDAR/VIS feature extraction.

In Formula (17), θ_*n*_ (*n* = 1, 2, ⋯ , *C*) represents the *n*th column parameter of the classifier, which *pred*(*i, j*) ∈ *R*^*C*^ is a one-dimensional vector, which represents the prediction result of the pixel *p*_*ij*_. For LiDAR or VIS data feature extraction, a cascaded CNN network is required, as shown in Figure 4.

From Figure 4, the cascade structure is mainly composed of basic cascade operations. Before entering the data into the network structure, it needs to be normalized. In the convolution operation, the convolution kernel size is set to 3 × 3. After going through the operations of all modules, expand the extracted feature through *F*_*LV*_ to obtain one-dimensional vector, and then use it as the input part of the fully connected layer. In order to improve the fusion effect of features at different levels, a Cascade block structure is designed in which different features can be bridged. This structure can be represented by Formula (18).


(18) $$\left\{ \begin{array}{l} y_{m}=g_{m}(x,\{W_{i},B_{i}\})+x\\ \;y=g_{s}(x_{s},\{W_{j},B_{j}\})+x_{s} \end{array}\right.$$

In Formula (18), *g*_*m*_ (*x*, {*W*_*i*_, *B*_*i*_})and *g*_*s*_ (*x*_*s*_, {*W*_*j*_, *B*_*j*_})is the operation between two channels, *x* and the *y* corresponding input and output, indicating the output of the middle layer. After the CNN network is constructed, all its parameters need to be trained and updated. For the network parameters, the feature map of each layer of the network is set to a power of 2. Since more parameters need to be trained and the distribution of these parameters is not uniform, training on two branches at the same time will have an impact on obtaining the optimal parameter solution. Therefore, it is necessary to train the parameters on the two branches separately, and then perform fine-tuning training after the two are trained. In training experiments, data and methods are the two most critical parts. Different from general deep learning training models, remote sensing image data training has a limited number of labels, and the labeling process is time-consuming and costly (Gu et al., 2022). To solve this problem, it is usually necessary to process the data in the preprocessing stage, such as rotating the image, adding Gaussian noise, etc., to expand the training set. In addition to this, all data needs to be normalized.

When performing feature extraction on HSI, 1-D CNN is responsible for extracting spectral features, while 2-D CNN is responsible for extracting spatial information (Xu et al., 2019). This dual-channel network design can reduce training update parameters, so it can save computing resources and improve training efficiency. In addition, the Cascade block structure also has certain advantages when extracting LiDAR/VIS data. This cascaded CNN network structure can transfer low-level features to high-level features, which can be reused to improve efficiency.

## 4. Performance analysis of multi source remote sensing data fusion and classification based on CNN

In order to effectively verify the performance of the proposed dual-channel CNN, the same type of classification models are introduced: SVM algorithm and ELM algorithm. During the performance analysis, the samples used by the three methods are the same. Use (H) to represent the experiments and results of the classification model on hyperspectral, and (H+L) to represent the experimental results of the combination of hyperspectral and LiDAR. First, the experimental results of DB-CNN network using different classification methods on different datasets are analyzed. The data sets involved are Houston data set, Trento data set, Pavia data set and Salinas data set. The Houston data set consists of two parts, namely hyperspectral data and LiDAR data. The map size is 349 ^*^ 1,905; Trento dataset is shot in Trento region, Italy, with 600 ^*^ 166 pixels; The Pavia dataset was taken in Pavia, Italy, with a map size of 610 ^*^ 340; The Salinas dataset was taken in the Salinas region of Italy, and the map size is 512 ^*^ 217. The analysis results are shown in Table 1.

**Table 1:** Comparison of classification accuracy of dual-branch CNN networks on different data sets.

**Data**	**DB-CNN(L/V)**		**DB-CNN(H)**		**DB-CNN(H**+**L/V)**	
	**OA (%)**	**Kappa**	**OA (%)**	**Kappa**	**OA (%)**	**Kappa**
Houston	55.62	0.5168	83.21	0.8157	86.69	0.8577
Trento	84.81	0.8105	94.98	0.9285	96.83	0.9547
Pavia	92.85	0.9042	96.87	0.9593	98.46	0.9735
Salinas	91.68	0.9107	95.53	0.9487	96.58	0.9576

**Figure 5:**
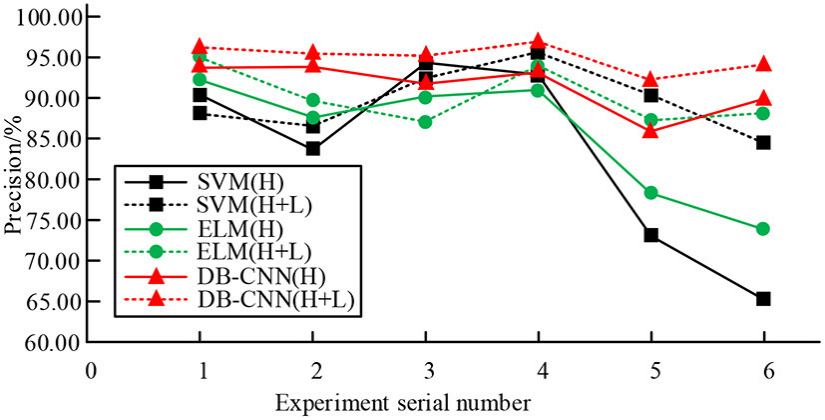
Comparison of classification accuracy of three classification models on Houston dataset.

In Table 1, compared with a single HSI or LiDAR method, the combined method has higher global classification accuracy in different data sets. For example, on the Pavia dataset, the global classification accuracy of the three classification methods is the highest, among which the global classification accuracy of the joint classification method reaches 98.46%, which is 5.61% higher than the single LiDAR/VIS classification accuracy and 1.59% higher than the single HSI classification accuracy. At the same time, the Kappa value of the classification accuracy index of the joint classification method is 0.9735, which is 0.0693 higher than the Kappa value of the single LiDAR/VIS classification and 0.0142 higher than the Kappa value of the single HSI classification. This result shows that the classification effect of the joint classification method is better than that of the single classification method. Classification method. At the same time, the Houston data set is taken as an example to verify the classification accuracy of different classification models on this data set. The comparison results are shown in Figure 5.

**Figure 6:**
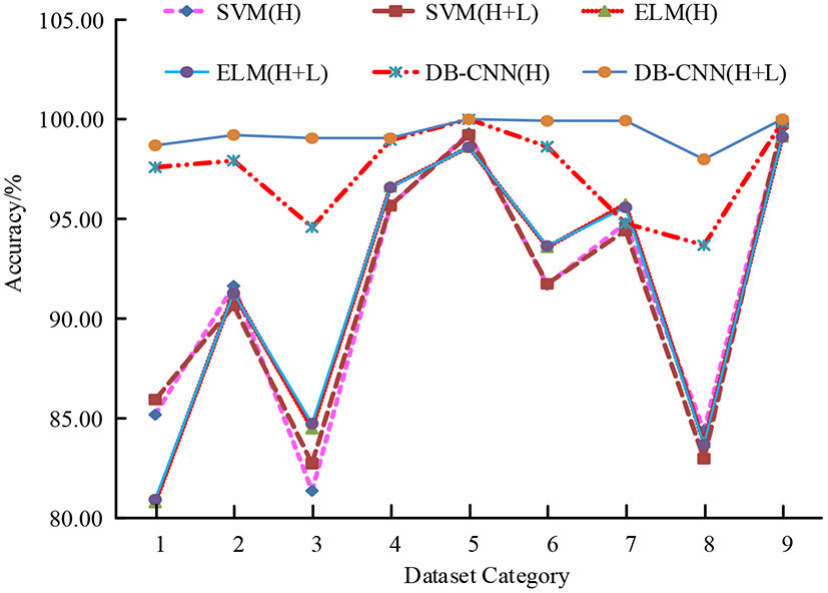
Accuracy of different classification models.

As can be seen from Figure 5, for the three classification models, the fusion classification method has the best performance and the highest classification accuracy in the global classification. For example, the average accuracy of SVM model using a single HSI classification is about 82.83%, and the average accuracy of SVM model using a combination of HSI and LiDAR classification is about 89.86%. The average accuracy of the ELM model using a single HSI classification is about 85.57%, and the average accuracy of the ELM model using a combination of HSI and LiDAR classification is about 91.05%. The average accuracy of DB-CNN model using a single HSI classification is about 92.13%, and the average accuracy of DB-CNN model using a combination of HSI and LiDAR classification is about 95.08%. Therefore, in the three classification models, the average classification accuracy of the single classification method and the joint classification method corresponding to the dual branch CNN network structure is higher than that of the SVM model and ELM model, indicating that the classification effect is better. The classification performance of the DB-CNN model is further analyzed through the Pavia dataset. The results are shown in Figure 6.

**Figure 7:**
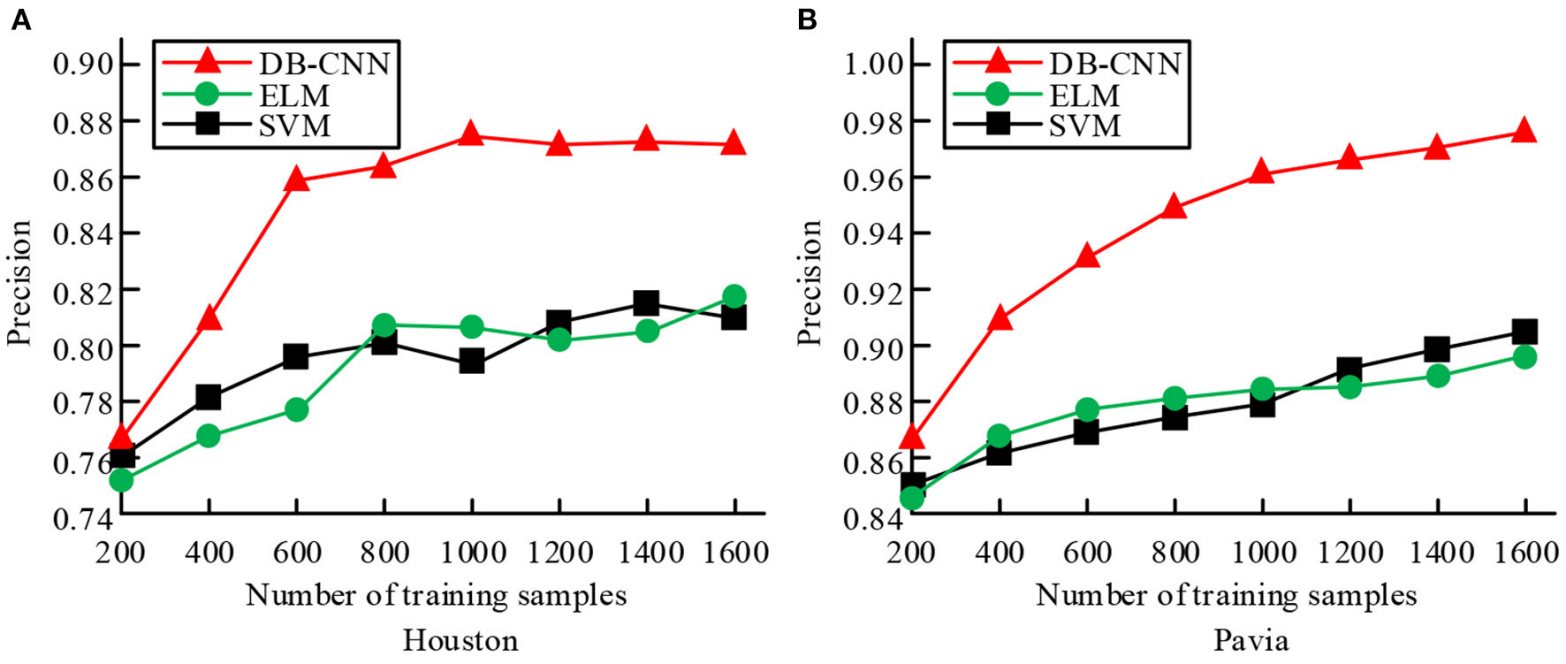
Comparison of classification performance of three classification models under different training sample numbers. **(A)** Training accuracy of the three classifications of Houston training set. **(B)** Training accuracy of the three classifications of Pavia training set.

In Figure 6, according to the trend of the broken line chart of the accuracy rate of the six classification models, compared with the classification models corresponding to the SVM algorithm and the ELM algorithm, the accuracy rate of the classification model corresponding to the DB-CNN is higher, especially the classification accuracy rate of the two branch CNN classification model is the highest, with the highest accuracy rate of 100.00%; Moreover, the accuracy of the two branch CNN classification model is above other models, and the accuracy difference between different data sets is small, that is, the performance of the two branch CNN classification model is more stable. In addition, the classification performance of the three classification models under different training sample numbers is compared, as shown in Figure 7.

Figure 7A shows the training accuracy of the three classifications of Houston training set, and Figure 7B shows the training accuracy of the three classifications of Pavia training set. According to the trend of the graph, in the process of increasing training samples, the classification accuracy of the three classification models shows an overall upward trend. Among them, the accuracy of the dual-branch CNN network model has an obvious upward trend, and its training accuracy is higher than the other two classification models under the same number of samples. And when the number of training samples is small, the dual-branch CNN network model can also achieve better classification accuracy. In Figure 7A, when the training sample size is 800, the accuracy of DB-CNN model is 0.862, 0.062 higher than that of SVM model; In Figure 7B, when the training sample size is 1,600, the precision of ELM model and DB-CNN model is 89.73 and 97.68, respectively. The results show that the two branch CNN network model can achieve better classification accuracy when performing correlation classification. The training and test times of the three classification models under different training and test sample numbers are compared, as shown in Table 2.

**Table 2:** Comparison of training time of three classification models under different training sample numbers.

**Number of training samples**	**Training time (s)**			**Test time (s)**		
	**SVM**	**ELM**	**DB-CNN**	**SVM**	**ELM**	**DB-CNN**
200	36.4	32.7	25.3	15.7	13.4	8.1
400	68.1	61.5	49.6	28.5	25.3	15.6
600	103.9	92.4	70.6	40.9	35.8	21.5
800	135.7	119.5	91.4	51.2	43.7	26.1
1,000	160.4	142.9	113.8	60.3	49.9	29.8

**Figure 8:**
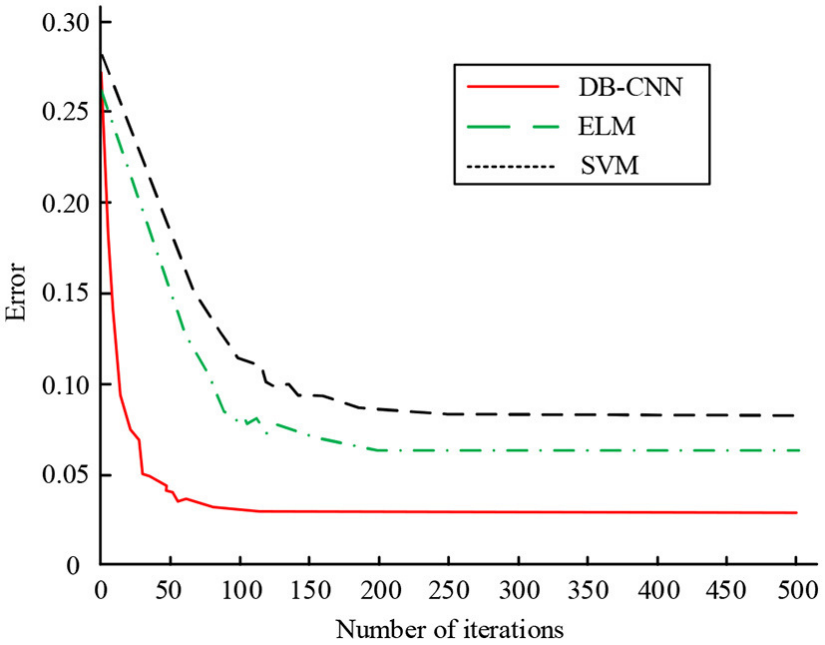
Test error comparison of three classification models on test set.

In Table 2, when the number of samples becomes large, the training time and testing time of the three classification models gradually increase, and the growth trend gradually slows down. When the number of samples used for training and testing is equal, the training and testing time of the dual-branch CNN network model is the shortest, followed by the ELM model, and the SVM model with the longest training and testing time. For example, when the number of samples used for training and testing is 1,000, the training time of the dual-branch CNN model is 113.8 s, which is 29.1 s lower than the ELM model and 46.6 s lower than the SVM model; its test time is 29.8 s, which is 20.1 s lower than the ELM model, which is 30.5 s lower than the SVM model. Therefore, under the same conditions, the training efficiency and testing efficiency of the dual-branch CNN network model are higher, and it has a better effect in the fusion and classification of multi-source remote sensing data. In addition, the test errors of the three classification models on the test set are compared and analyzed, as shown in Figure 8.

**Figure 9:**
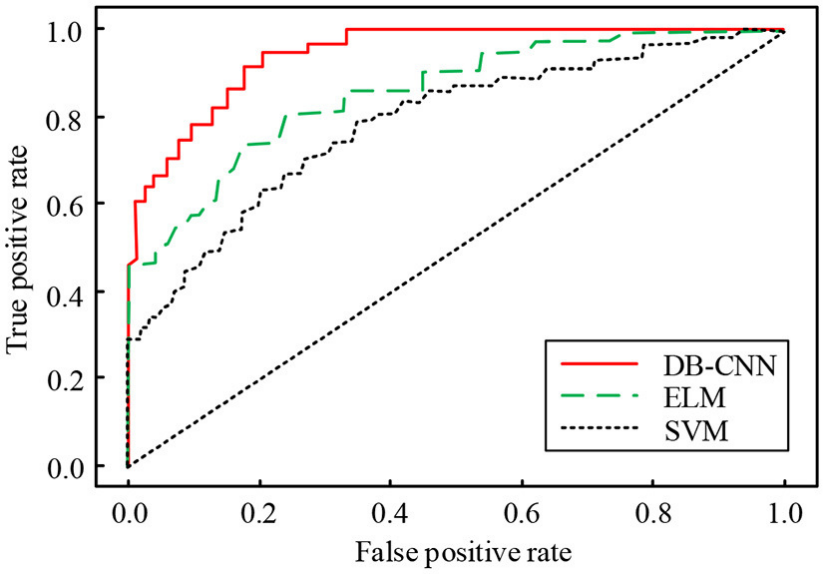
Comparison of ROC curves of three classification models.

In Figure 8, as the number of iterations increases, the classification errors of the three models gradually decrease and finally become stable. When the number of iterations is at a small level, the convergence speed of the dual-branch CNN network model was faster, followed by the ELM model and the SVM model. At 100 iterations, the error value of the dual-branch CNN network model is minimized and stabilized, and its error value is about 0.026. At 200 iterations, the error value of the dual-branch CNN network model is minimized and stabilized, the error value of the ELM model is minimized and stabilized, and its error value is about 0.063, the dual branch CNN network model is 0.100. When the number of iterations reaches 200, the error value of the SVM model decreases to a minimum and tends to be stable. According to the results, the two branch CNN network model has the smallest error value and the best classification effect. Finally, the ROC curves of the three classification models are compared, as shown in Figure 9.

In Figure 9, the lower area corresponding to the ROC curve of the dual-branch CNN network model is the largest, that is, the AUC value is the largest, followed by the ELM model and the SVM model. The AUC value corresponding to the dual-branch CNN network model is about 0.922. AUC value of ELM model is about 0.869, which is 0.053 lower than the dual-branch CNN network model. AUC value of SVM model is about 0.837, which is 0.032 lower than the ELM model and 0.085 lower than the dual-branch CNN network model. The ROC curve and AUC value represent the quality of the classification effect. From the above results, we can see that the classification effect of the dual-branch CNN network model is the best, and it can play a greater role in the recognition and classification of remote sensing images.

## 5. Conclusion

CNN can better classify and recognize, and they have been widely used in many fields. In order to realize the fusion and classification of multi-source remote sensing data, a dual branch CNN network structure model is proposed, and ELM model and SVM model are used as comparison models. According to the results obtained, it can be seen that for the dual branch CNN network, the HSI and LiDAR joint classification method has the highest global classification accuracy on different data sets. On the Pavia dataset, the global classification accuracy of the three classification methods is the highest. Among them, the global classification accuracy of the joint classification method is 98.46, 5.61% higher than that of the single LiDAR/VIS classification, and 1.59% higher than that of the single HSI classification. In the training experiment, compared with other methods, the training accuracy of BD-CNN model is higher than that of the other two classification models with the same sample number. When the number of samples used in training and testing is the same, the training time and testing time of BD-CNN model are the lowest. In the error test experiment, when the number of iterations of the DB-CNN model is 100, the test error reaches the lowest steady state, which is about 0.026, 0.037 lower than the ELM model. In addition, the ROC curve of the DB-CNN model corresponds to the largest lower area, that is, the AUC value is the largest, which is about 0.922, that is, the DB-CNN model has the best classification performance. Comprehensive analysis shows that BD-CNN model can effectively fuse and classify multi-source remote sensing data. However, there is still room for improvement. In this paper, we can discuss other depth learning methods when classifying remote sensing data to obtain better classification results.

## Data availability statement

The original contributions presented in the study are included in the article/supplementary material, further inquiries can be directed to the corresponding author.

## Author contributions

FY and ZZ contributed to conception and design of the study. YW organized the database. BE performed the statistical analysis. FY wrote the first draft of the manuscript. ZZ wrote sections of the manuscript. All authors contributed to manuscript revision, read, and approved the submitted version.

## References

[B1] Bazi Y. Bashmal L. Rahhal M. M. A. Dayil R. A. Ajlan N. A. (2021). Vision transformers for remote sensing image classification. Remote Sens. 13, 516–521. 10.3390/rs1303051636104442

[B2] Chung Y. L. Chung H. Y. Tsai W. F. (2020). Hand gesture recognition *via* image processing techniques and deep CNN. J. Intell. Fuzzy Syst. 39, 1–14. 10.3233/JIFS-20038535618260

[B3] Demir V. Ulke A. (2020). Obtaining the manning roughness with terrestrial-remote sensing technique and flood modeling using FLO-2D: a case study Samsun from Turkey. Geofizika 37, 131–156. 10.15233/gfz.2020.37.9

[B4] Deng Z. Cao Y. Zhou X. Yi Y. Jiang Y. You I. (2020). Toward efficient image recognition in sensor-based IoT: a weight initialization optimizing method for CNN based on RGB influence proportion. Sensors, 20, 2866–2871. 10.3390/s20102866 32443591 PMC7288215

[B5] Dong R. Xu D. Jiao L. Zhao J. An J. (2020). A fast deep perception network for remote sensing scene classification. Remote Sens. 12, 422–439. 10.3390/rs12040729

[B6] Dong Y. Liang T. Zhang Y. Du B. (2020). Spectral-spatial weighted kernel manifold embedded distribution alignment for remote sensing image classification. IEEE Trans. Cybernet. 51, 3185–3197. 10.1109/TCYB.2020.3004263 32649289

[B7] Du X. Zheng X. Lu X. Doudkin A. A. (2021). Multisource remote sensing data classification with graph fusion network. IEEE Trans. Geosci. Remote Sens. 59, 10062–10072. 10.1109/TGRS.2020.3047130

[B8] Du Y. Qin B. Zhao C. Zhu Y. Cao J. Ji Y. (2022). A novel spatio-temporal synchronization method of roadside asynchronous MMW radar-camera for sensor fusion. IEEE Trans. Intell. Transp. Syst. 23, 22278–22289. 10.1109/TITS.2021.3119079

[B9] Feng Z. Zhu M. Stanković L. Ji H. (2021). Self-matching CAM: a novel accurate visual explanation of CNNs for SAR image interpretation. Remote Sens. 13, 1772–1778. 10.3390/rs13091772

[B10] Gu X. Zhang C. Shen Q. Han J. Angelov P. P. Atkinson P. M. (2022). A Self-training hierarchical prototype-based ensemble framework for remote sensing scene classification. Inform. Fusion 80, 179–204. 10.1016/j.inffus.2021.11.014

[B11] Han Y. Wei C. Zhou R. Hong Z. Zhang Y. Yang S. (2020). Combining 3D-CNN and squeeze-and-excitation networks for remote sensing sea ice image classification. Math. Probl. Eng. 2020, 8065396. 10.1155/2020/8065396

[B12] He C. He B. Tu M. Wang Y. Qu T. Wang D.. (2020). Fully convolutional networks and a manifold graph embedding-based algorithm for PolSAR image classification. Remote Sens. 12, 1467–1473. 10.3390/rs12091467

[B13] Hu A. Chen S. Wu L. Xie Z. Qiu Q. Xu Y. (2021). WSGAN: an improved generative adversarial network for remote sensing image road network extraction by weakly supervised processing. Remote Sens. 13, 2506–2511. 10.3390/rs13132506

[B14] Huang C. Q. Jiang F. Huang Q. H. Wang X. Z. Han Z. M. Huang W. Y. (2022). Dual-graph attention convolution network for 3-d point cloud classification. IEEE Trans. Neural Netw. Learn. Syst. 2022, 1–13. 10.1109/TNNLS.2022.3162301 35385393

[B15] Jin H. Mountrakis G. (2022). Fusion of optical, radar and waveform LiDAR observations for land cover classification. ISPRS J. Photogram. Remote Sens. 187, 171–190. 10.1016/j.isprsjprs.2022.03.010

[B16] Li Z. Zhou A. Shen Y. (2020). An end-to-end trainable multi-column CNN for scene recognition in extremely changing environment. Sensors 20, 1556–1562. 10.3390/s20061556 32168843 PMC7147165

[B17] Lu H. Zhu Y. Yin M. Yin G. Xie L. (2022). Multimodal fusion convolutional neural network with cross-attention mechanism for internal defect detection of magnetic tile. IEEE Access 10, 60876–60886. 10.1109/ACCESS.2022.3180725

[B18] Luo X. Du H. Zhou G. Li X. Mao F. Zhu D. E.. (2021). A novel query strategy-based rank batch-mode active learning method for high-resolution remote sensing image classification. Remote Sens. 13, 2234–2256. 10.3390/rs13112234

[B19] Ma H. Liu Y. Ren Y. Wang D. Yu L. Yu J. (2020). Improved CNN classification method for groups of buildings damaged by earthquake, based on high resolution remote sensing images. Remote Sens. 12, 260. 10.3390/rs12020260

[B20] Pan X. Zhao J. Xu J. (2020). An end-to-end and localized post-processing method for correcting high-resolution remote sensing classification result images. Remote Sens. 12, 852–856. 10.3390/rs12050852

[B21] Pastorino M. Montaldo A. Fronda L. Hedhli I. Moser G. Serpico S. B.. (2021). Multisensor and multiresolution remote sensing image classification through a causal hierarchical markov framework and decision tree ensembles. Remote Sens. 13, 849–874. 10.3390/rs13050849

[B22] Qing Y. Liu W. Feng L. Gao W. (2021). Improved transformer net for hyperspectral image classification. Remote Sens. 13, 2216–2220. 10.3390/rs13112216

[B23] Qu L. Zhu X. Zheng J. Zou L. (2021). Triple-attention-based parallel network for hyperspectral image classification. Remote Sens. 13, 324–329. 10.3390/rs13020324

[B24] Samat A. Li E. Wang W. Liu S. Lin C. Abuduwaili J. (2020). Meta-XGBoost for hyperspectral image classification using extended MSER-guided morphological profiles. Remote Sens. 12, 1973–1978. 10.3390/rs12121973

[B25] Sun Z. Liu M. Liu P. Li J. Yu T. Gu X.. (2021). SAR image classification using fully connected conditional random fields combined with deep learning and superpixel boundary constraint. Remote Sens. 13, 271–278. 10.3390/rs13020271

[B26] Xu Y. Du B. Zhang L. Cerra D. Pato M. Carmona E.. (2019). Advanced multi-sensor optical remote sensing for urban land use and land cover classification: outcome of the 2018 IEEE GRSS data fusion contest. IEEE J. Select. Topics Appl. Earth Observ. Remote Sens. 12, 1709–1724. 10.1109/JSTARS.2019.2911113

[B27] Xu Z. Zhao X. Guo X. Guo J. (2019). Deep learning application for predicting soil organic matter content by VIS-NIR spectroscopy. Comput. Intell. Neurosci. 2019, 1–11. 10.1155/2019/3563761

[B28] Yu D, Xu, Q, Guo, H, Zhao, C, Lin, Y, Li, D. (2020). An efficient and lightweight convolutional neural network for remote sensing image scene classification. Sensors 20, 1999–2005. 10.3390/s20071999 32252483 PMC7181261

[B29] Zhang C. Chen Y. Yang X. Gao S. Li F. Kong A.. (2020). Improved remote sensing image classification based on multi-scale feature fusion. Remote Sens. 12, 213–219. 10.3390/rs12020213

[B30] Zhang H. Han J. (2020). Mathematical models for information classification and recognition of multi-target optical remote sensing images. Open Phys. 18, 951–960. 10.1515/phys-2020-0123

[B31] Zhang Q. Ge L. Hensley S. Metternicht G. I. Liu C. Zhang R (2022). PolGAN: a deep-learning-based unsupervised forest height estimation based n the synergy of PolInSAR and LiDAR data. ISPRS J. Photogram. Remote Sens. 186, 123–139. 10.1016/j.isprsjprs.2022.02.008

[B32] Zhang W. He X. Lu W. (2020). Exploring discriminative representations for image emotion recognition with CNNs. IEEE Trans. Multimedia, 22, 515–523. 10.1109/TMM.2019.2928998

[B33] Zhong T. Cheng M. Lu S. Dong X. Li Y. (2022). RCEN: a deep-learning-based background noise suppression method for DAS-VSP records. IEEE Geosci. Remote Sens. Lett. 19, 1–5. 10.1109/LGRS.2021.3127637

[B34] Zhou G. Li C. Zhang D. Liu D. Zhou X. Zhan J. (2021a). Overview of underwater transmission characteristics of oceanic LiDAR. IEEE J. Select. Topics Appl. Earth Observ. Remote Sens. 14, 8144–8159. 10.1109/JSTARS.2021.3100395

[B35] Zhou G. Li W. Zhou X. Tan Y. Lin G. Li X.. (2021b). An innovative echo detection system with STM32 gated and PMT adjustable gain for airborne LiDAR. Int. J. Remote Sens. 42, 9187–9211. 10.1080/01431161.2021.1975844

[B36] Zhou G. Zhou X. Song Y. Xie D. Wang L. Yan G. (2021c). Design of supercontinuum laser hyperspectral light detection and ranging (LiDAR) (SCLaHS LiDAR). Int. J. Remote Sens. 42, 3731–3755. 10.1080/01431161.2021.1880662

[B37] Zhou W. Wang H. Wan Z. (2022). Ore image classification based on improved CNN. Comput. Electrical Eng. 99, 107819. 10.1016/j.compeleceng.2022.107819

